# Highly sensitive AlGaN/GaN HEMT biosensors using an ethanolamine modification strategy for bioassay applications†

**DOI:** 10.1039/c9ra02055a

**Published:** 2019-05-16

**Authors:** Zhiqi Gu, Jin Wang, Bin Miao, Lei Zhao, Xinsheng Liu, Dongmin Wu, Jiadong Li

**Affiliations:** School of Nano Technology and Nano Bionics, University of Science and Technology of China Hefei 230026 China; i-Lab, Suzhou Institute of Nano-Tech and Nano-Bionics, Chinese Academy of Sciences Suzhou 215125 People's Republic of China jdli2009@sinano.ac.cn; Key Laboratory of Multifunctional Nanomaterials and Smart Systems, Chinese Academy of Sciences Suzhou 215125 People's Republic of China; The College of Materials Sciences and Engineering, Shanghai University Shanghai 200444 China; The College of Nuclear Technology and Automation Engineering, Chengdu University of Technology Chengdu 610059 China

## Abstract

In this paper, we propose a highly efficient surface modification strategy on an AlGaN/GaN high electron mobility transistor (HEMT), where ethanolamine (EA) was utilized to functionalize the surface of GaN and provided amphoteric amine groups for probe molecular immobilization for bioassay application. The molecular gated-AlGaN/GaN HEMT was utilized for pH and prostate-specific antigen (PSA) detection to verify its performance as a biosensor. Benefitting from the high coating quality on the GaN surface, the performance of our biosensor is drastically improved compared to other AlGaN/GaN HEMT based pH and PSA biosensors reported before. Our molecular gated-AlGaN/GaN HEMT biosensor has achieved good static electrical performance for pH sensing, such as high sensitivity, good linearity and chemical stability. Moreover, after further immobilization of PSA antibody onto the EA aminated GaN surface, the limit of detection (LOD) for PSA detection is as low as 1 fg mL^−1^ in PBS buffer, which has reached an at least two orders of magnitude decrease compared to any other AlGaN/GaN HEMT based PSA biosensor reported before. And the sensitivity of our PSA biosensor has achieved a substantial increase, reaching up to 2.04% for 100 ng mL^−1^. The measurements of pH and PSA utilizing the EA modified AlGaN/GaN HEMT biosensor indicate that the surface modification strategy on the GaN proposed in this paper can effectively improve the performance of the AlGaN/GaN HEMT based biosensor, which demonstrates a promising application prospect in the AlGaN/GaN HEMT based biological detection field.

## Abstract 2

We propose a highly efficient surface modification strategy on an AlGaN/GaN high electron mobility transistor, where ethanolamine was utilized to functionalize the surface of GaN and provided amphoteric amine groups for bioassay application.

## Graphical abstract

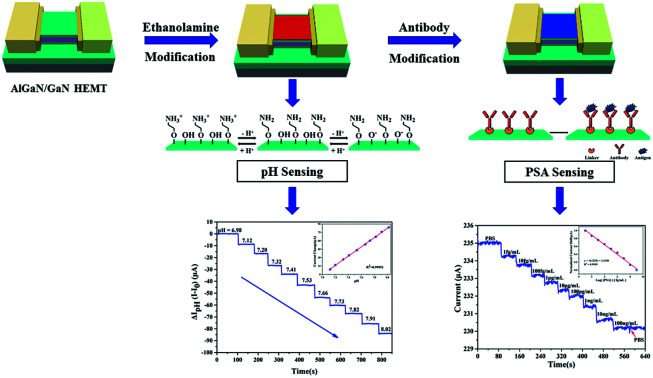

## 1. Introduction

Tremendous efforts in the efficient detection of biological analytes are being made due to significant applications in environmental monitoring, disease diagnosis and food safety, among others. Biological sensors, viewed as a part of bioanalytical technology, were proposed to quickly analyze and track biological analytes that need to be monitored, such as optical biosensors,^1^ electrochemical biosensors,^2^ calorimetric biosensors^3^ and so on. Among various biological sensors, semiconductor biosensors have been widely researched for their advantages in miniaturization, integration and multifunction.^4–6^

A third generation semiconductor material, represented by wide band gap gallium nitride (GaN), was considered to possess broad application prospects in the field of biosensors for its excellent material properties.^7–9^ An important application of GaN in the field of biosensors is AlGaN/GaN HEMTs for its good biocompatibility, stable material properties and high sensitivity to the surface charge changes since the two-dimensional electron gas (2DEG) channel is well close to the surface. Up to now, many studies have been conducted on AlGaN/GaN HEMT to detect different biological analytes. Eickhoff *et al.* firstly utilized AlGaN/GaN HEMT for pH detection while oxidized GaN surface served as the sensing membrane to interact with hydrogen ion.^10^ Fan *et al.* used Au as the gate of AlGaN/GaN HEMT, which broadened the types of detection based on AlGaN/GaN HEMT biosensors, and have successfully realized the detection of ions,^11^ protein^12^ and DNA.^13^ Moreover, they proposed some inorganic materials as sensing membranes and further extended the application range of AlGaN/GaN HEMT biosensors while improved sensitivity to some extent.^14–16^ However, the high-energy deposition progresses of Au and those inorganic materials would induce native oxide and finally result in device instability and reducing in response performance.^17^ To overcome these problems, our team developed molecular gated-AlGaN/GaN HEMT biosensors and have verified their feasibility in Hg^2+^,^18^ prostate-specific antigen (PSA),^19^ TNT^20^ and pH^21^ detection. Although they have shown good performance, their relatively low sensitivity still limits their application in actual detection.

Recently, Ebner *et al.* proposed a new surface amination method on silicon nitride AFM tips and the modification efficiency was well improved where ethanolamine (EA) was utilized for surface amine-functionalization.^22^ Janissen *et al.* also reported a new modification method utilizing EA on InP nanowires, in their work EA has been proven to effectively improve the sensitivity of InP based field-effect transistor (FET) biosensor for that it can provide highly homogeneous and dense amine-coating than that of 3-aminopropyltriethoxysilane (APTES) modification.^23^ However, it is still unclear whether EA is feasible to be applied on GaN. In this work, we firstly verified the feasibility of modifying EA on GaN, and further the molecular gated-AlGaN/GaN HEMT biosensors using EA modification are proven to significantly increase their sensitivity in pH detection and PSA detection (by subsequent modification), and it is reasonable to believe EA modification can also do help to improve the performance of AlGaN/GaN HEMT biosensors for other bioassay applications.

## 2. Material and methods

### 2.1. Reagents

Ethanolamine hydrochloride, which was utilized for GaN surface modification, was purchased from Aladdin (Shanghai, China), and the water free solvent dimethyl sulfoxide (DMSO) was from Sinopharm Chemical Reagent Co, Ltd. Reagents for preparing buffer for pH detection, including sulfuric acid (H_2_SO_4_), sodium sulfate (Na_2_SO_4_), sodium bicarbonate (NaHCO_3_), sodium carbonate (Na_2_CO_3_), sodium dihydrogen phosphate (NaH_2_PO_4_), sodium hydrogen phosphate (Na_2_HPO_4_) were bought from Sinopharm Chemical Reagent Co, Ltd. The glutaraldehyde solution used for PSA antibody modification and the bovine serum albumin (BSA) which was utilized to block the gate region to avoid nonspecific binding of PSA were purchased from Sigma-Aldrich Co. LLC. (Shanghai, China), and the PSA antibody and antigen were provided by Abcam. Unless stated otherwise, all other chemicals were of commercially available analytical regent grade. The gold nanoparticles (Au NPs) with the average diameter of 50 nm was purchased from Nanjing XFNANO Materials Tech Co., Ltd (Nanjing, China). Deionized water used in this work all purified using Milli-Q water purification.

### 2.2. Microfabrication of AlGaN/GaN high electron mobility transistor (HEMT)

**Fig. 1:**
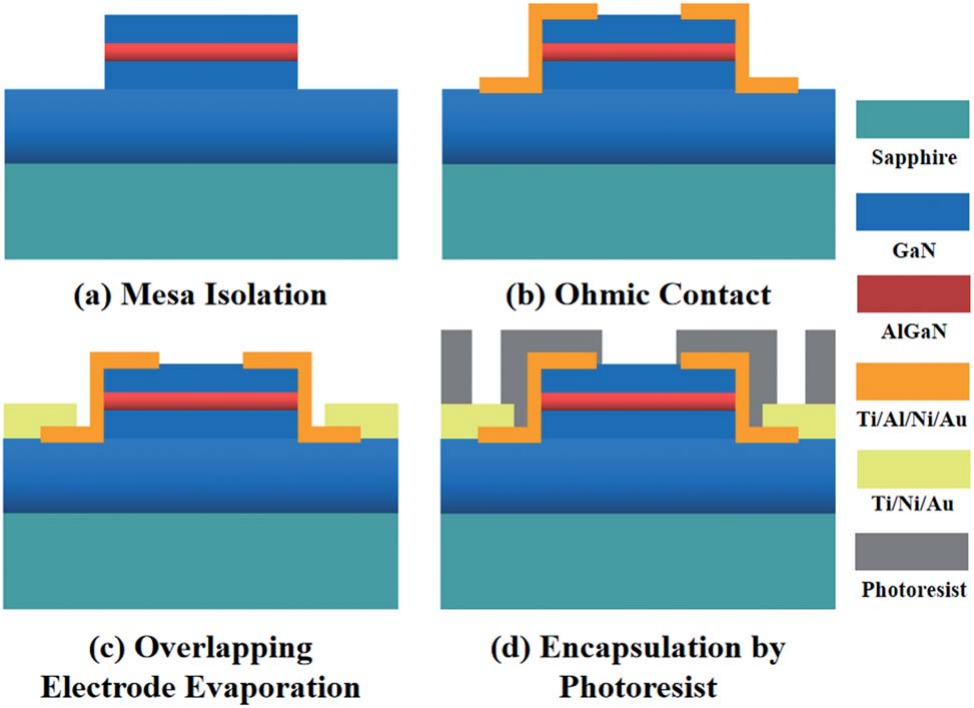
Schematic illustration of the fabrication process of the AlGaN/GaN HEMT biosensor.

The AlGaN/GaN HEMT structure studied in this work were grown on a sapphire substrate, which is composed of a 1.5 μm-thick GaN buffer layer, an 18 nm-thick AlGaN barrier layer, and a 1.5 nm-thick GaN cap layer. The specific fabrication progress is described in Fig. 1. Firstly, the mesa isolation was formed by inductively coupled plasma (ICP) etching with Cl_2_/BCl_3_ gases. Ohmic contacts were created by e-beam deposition of a Ti/Al/Ni/Au = 200 Å/1200 Å/700 Å/1000 Å multilayer metal structure, after which a rapid thermal annealing in nitrogen environment at 880 °C for 45 seconds was conducted. An overlapping multilayer of Ti/Ni/Au = 200 Å/700 Å/1000 Å was later evaporated above the ohmic contact to form electrodes. Photoresist was encapsulated onto the wafer, then the wafer was diced into units and mounted on the printed circuit board (PCB). Finally, the electrodes and the pads of PCB were bonded with gold wire to form the sensor units. The photograph of the AlGaN/GaN HEMT sensor is shown in Fig. S1.†

### 2.3. Modification of molecular-gated AlGaN/GaN HEMT biosensors

To modify AlGaN/GaN HEMT with EA, following steps were performed. Firstly, to guarantee a clean oxide sensing surface, the device was treated in a UV/O_3_ chamber (400 W, 10 min), after which the sensing membrane was immersed in 5 M ethanolamine hydrochloride in water free DMSO for 12 h at room temperature for homogeneous amination. The recipe we employed here was based on former studies where the influence of molarities and temperatures were discussed.^24^ After the progress of amination, the sensing membrane was washed with dry DMSO, twice with pure ethanol and finally rinsed with Milli-Q water and dried again in a nitrogen flow.

To further evaluate the performance for PSA antigen detection, the sensor was immersed in a glutaraldehyde solution with the concentration of 1.25% (v/v) for 2 h at room temperature to form a Schiff base after the modification of EA. A phosphate buffer saline solution (PBS, prepared with 1.8 mM KH_2_PO_4_, 10 mM Na_2_HPO_4_, 137 mM NaCl and 2.7 mM KCl, pH = 7.4) containing 10 μg mL^−1^ of anti-PSA was introduced onto the sensing membrane at 4 °C for 24 h, in order to anchor the anti-PSA antibody on the surface of gate through the carboxyl functional group. Finally, the gate region was blocked with 1% (v/v) bovine serum albumin (BSA) solution to avoid nonspecific binding of PSA.

### 2.4. Measurements of AlGaN/GaN HEMT biosensors

The response of the source and drain current (*I*_ds_) at fixed source and drain voltage (*V*_ds_) of EA modified AlGaN/GaN HEMT to change in the solution pH was evaluated by fabricated a cell consisting of a micro-chamber between a poly (dimethylsiloxane) (PDMS) mold and the AlGaN/GaN HEMT. All devices were soaked in deionized water for 12 hours before measurements to improve hydrolytic stability.^25^ To minimize device-to-device response variability, current change (*I* − *I*_0_) was used to represent the response of our sensor.

For PSA detection, after successively functionalized progress for PSA antibody modification, the PBS containing PSA at a concentration range of 1 fg mL^−1^ to 100 ng mL^−1^ were added to the sensing chamber. AlGaN/GaN HEMT biosensors were connected to a Keithley 2636A for data acquisition, while the source and drain voltage was fixed at 200 mV and the sampling interval is set at 1 s. All measurements were performed at room temperature.

## 3. Result and discussion

### 3.1. Characterization of the EA modification

**Fig. 2:**
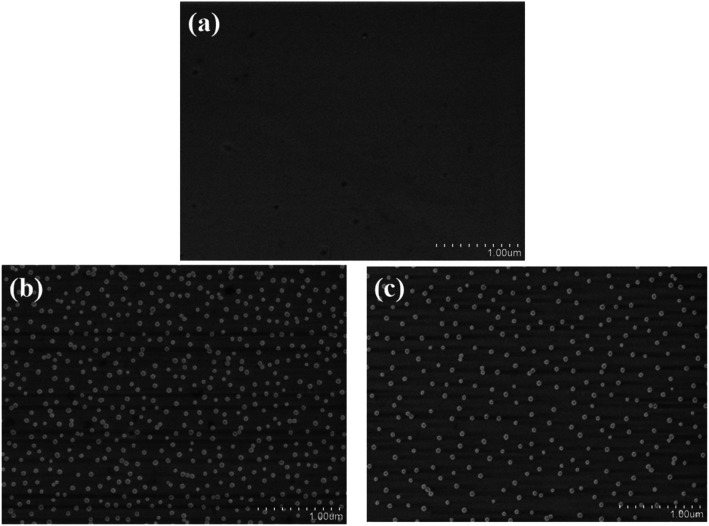
SEM images of GaN surface immersed in an AuNPs aqueous solution. (a) Bare surface; surface modified by EA (b) and surface modified by APTES (c).

To verify the immobilization of EA on GaN surface and to compare the difference in modification efficiency between EA and APTES, gold nanoparticles (Au NPs) was utilized because there exists a stable bond between amine group and the surface of Au NPs.^26^ It can be observed in Fig. 2 that both EA and APTES modification can covalently link amines onto the surface of GaN. The density of Au NPs on the EA modified surface (Fig. 2b) is higher than that of APTES modified one (Fig. 2c), which means EA modified surface could provide more active sites and more opportunities for bonding of biomolecular and therefore enhance the sensitivity of our biosensor.

### 3.2. The feasibility of EA modified AlGaN/GaN HEMT biosensors in bioassay application

#### 3.2.1 pH detection

**Fig. 3:**
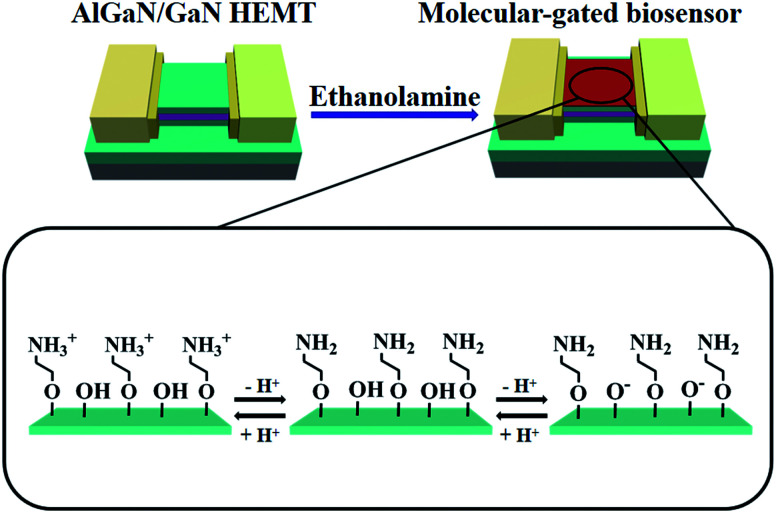
Schematic illustrating the conversion of AlGaN/GaN HEMT into a molecular-gated nanosensor for pH sensing. The surface of GaN was coated by amine by EA modification, zoom of the amine coated GaN surface illustrating changes in the surface charge state with pH.

Since pH detection is relatively simple in bioassay application, pH detection could be of our primary choice to verify the performance of our AlGaN/GaN HEMT biosensors. We first illustrated the basic concept of our experiment for the case of a pH sensor in Fig. 3. The principle of pH detection can be understood by considering the mixed surface functionality of the modified AlGaN/GaN HEMT. Covalently linking EA to GaN oxide surface resulted in a surface terminating in both [–NH_2_] and [–OH] groups. When pH increases by changing the composition of the buffer, the [–OH] group was deprotonated to [–O^−^] and the original [–NH_3_^+^] would lose proton to [–NH_2_]. In contrast, the [–NH_2_] group and the original [–O^−^] group would protonate to [–NH_3_^+^] and [–OH] when pH decrease. These protonation and deprotonation progress mentioned above would positively or negatively change the surface potential at the interface between the GaN surface and electrolyte.

Since AlGaN/GaN HEMT is extremely sensitive to the change of surface potential, the potential change will be coupled with 2DEG in the channel of AlGaN/GaN HEMT, finally change the source and drain current, which can be described as:^18^1
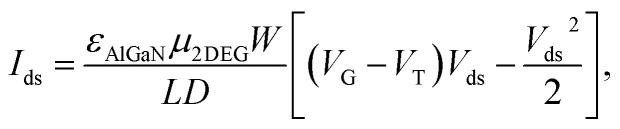
where *ε*_AlGaN_ is the permittivity of AlGaN, *μ*_2DEG_ is electron mobility of the 2DEG, *W* and *L* represent the width and length of the channel, *D* means the distance between the 2DEG channel and the surface, *V*_G_ and *V*_T_ and *V*_ds_ represent the gate voltage, threshold voltage and source and drain voltage, respectively. Therefore, the changes in the surface potential of GaN possess the ability to cause changes in source and drain current (*I*_ds_) of AlGaN/GaN HEMT.

**Fig. 4:**
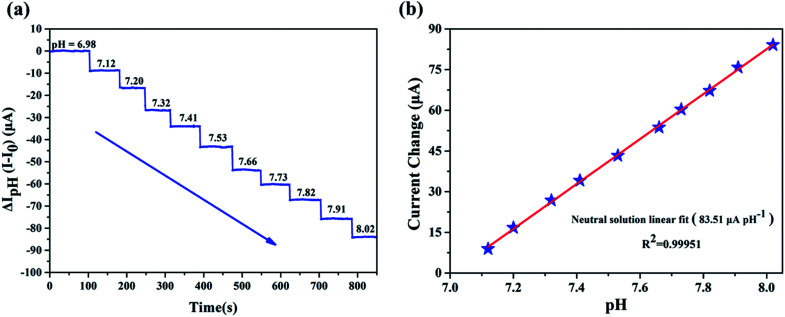
EA modified AlGaN/GaN HEMT for pH detection in the range 6.98–8.02, the step is 0.1 pH, calibrated with a commercial pH meter. (a) Detection of the neutral solution by mixing different volumes of 0.2 M NaH_2_PO_4_ solution and 0.2 M Na_2_HPO_4_ solution. The sensitivity is 83.51 μA pH^−1^. (b) The linearity for pH detection in pH = 6.98–8.02.

We evaluated the performance of our biosensor in neutral solution (pH = 6.98–8.02), which is considered to be the range relevant for human blood.^15^Fig. 4a illustrated the clear change of source and drain current (*I*_ds_), measurements of source and drain current (*I*_ds_) as a function of time demonstrate that *I*_ds_ decreased stepwise along with the discrete change of buffer's pH and stays constant for a given pH without any further treatment. The precise pH measured by a commercial digital pH meter with precision of 0.01 was marked out in the Fig. 4a. The corresponding plot of the *I*_ds_*versus* pH presented in Fig. 4b indicates that our pH sensor based on EA modified AlGaN/GaN HEMT possesses pretty linearity for pH dependence characteristic and its calculated sensitivity reached up to 83.51 μA pH^−1^, the standard deviation of the test is 2.79%.

**Fig. 5:**
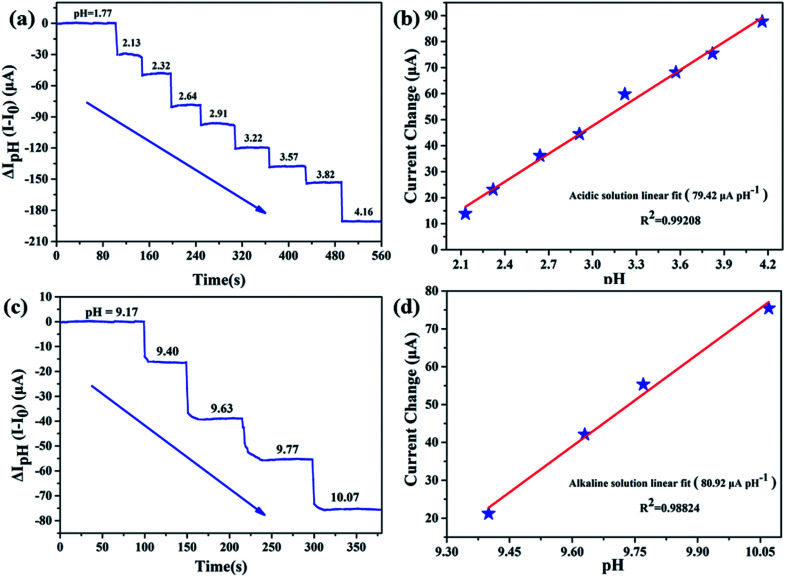
Source and drain current (*I*_ds_) responses of EA modified AlGaN/GaN HEMT for pH detection. (a) Detection of the acidic solution by mixing different volumes of 0.01 M H_2_SO_4_ and 0.01 M Na_2_SO_4_. The sensitivity is 79.42 μA pH^−1^. (b) The corresponding linearity for pH detection in acidic solution. (c) Detection of the alkaline solution by mixing different volumes of 0.1 M NaHCO_3_ and 0.1 M Na_2_CO_3_. The sensitivity is 80.92 μA pH^−1^. And (d) the corresponding linearity for pH detection in alkaline solution.

In our previous work, we have proven that APTES modified AlGaN/GaN HEMT pH sensor can effectively detect pH in acidic and alkaline solutions.^21^ To validate whether EA modified AlGaN/GaN HEMT pH sensor can also keep stable pH detection performance in those environments, we conducted the pH detection in the range of pH = 1.77–4.16 and pH = 9.17–10.07. Measurements of *I*_ds_ as function of time are shown in Fig. 5a (in acidic solution) and Fig. 5c (in alkaline solution), respectively. The sensitivities of our biosensor are calculated as 79.42 μA pH^−1^ for acidic solution and 80.92 μA pH^−1^ for alkaline solution. A typical plot of the *I*_ds_*versus* pH demonstrates that this pH dependence is linear in acidic (Fig. 5b) and alkaline solution (Fig. 5d), and the standard deviations of the test are 2.12% (for pH = 1.77–4.16) and 2.53% (for pH = 9.17–10.07).

To conclude, our biosensor exhibits good sensitivity and corresponding linearity performance in acidic, neutral and alkaline solution, which suggests that EA modified AlGaN/GaN HEMT could function as a microscale pH sensor.

#### 3.2.2 Prostate-specific antigen (PSA) detection

**Fig. 6:**
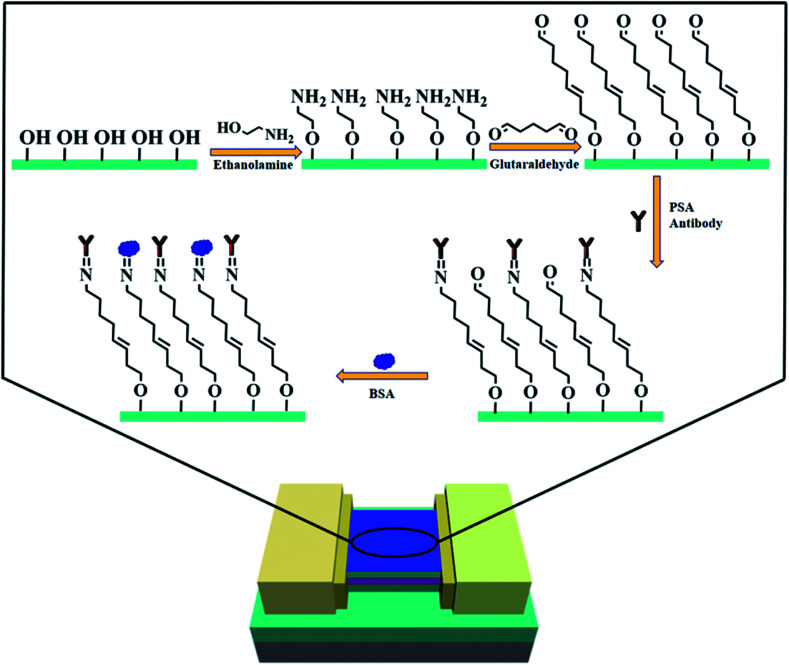
Schematic illustration of the entire PSA antibody modification process on AlGaN/GaN HEMT.

Since EA modified AlGaN/GaN HEMT biosensor has proven to be a qualified pH sensor, we would further explore the feasibility of biomolecular sensing. PSA was chosen as the target detection for which was considered to be the best biomarker for prostate cancer and a typical protein.^27^ On the basis of the EA modified AlGaN/GaN HEMT biosensor, we successively functionalize the sensing membrane with glutaraldehyde and PSA antibody, finally bovine serum albumin (BSA) solution with the concentration of 1% (v/v) was added to avoid nonspecific binding of PSA, the entire progress was presented in Fig. 6.

**Fig. 7:**
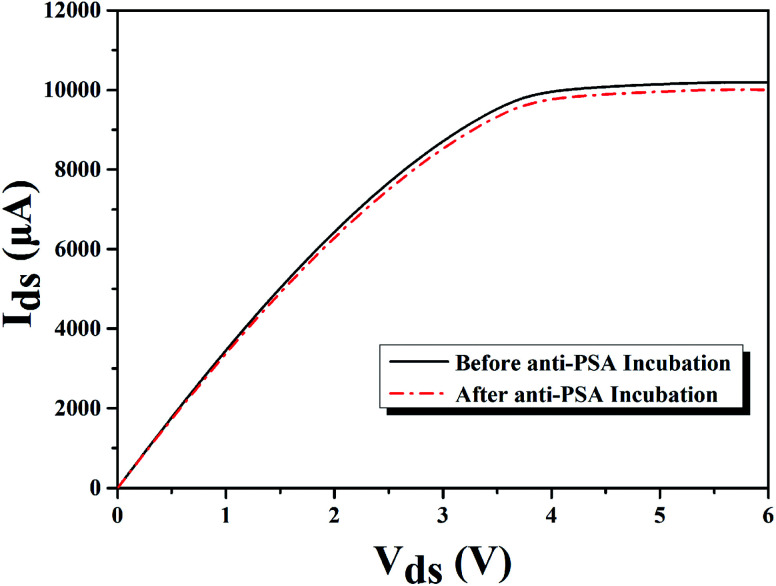
*I*–*V* characteristics of molecular modified AlGaN/GaN HEMT biosensor before and after anti-PSA incubation for PSA detection.

After the sensing region of our AlGaN/GaN HEMT based biosensor was well modified, the PBS (phosphate buffer saline) solution containing PSA antibody (anti-PSA) at a concentration of 10 μg mL^−1^ was added to the sensing chamber, then the sensing region was rinsed with deionized water and dried in nitrogen flow. The source and drain current of the AlGaN/GaN HEMT based biosensor were measured before and after anti-PSA incubation, as is exhibited in Fig. 7. The changes in the surface charge originating from the covalent coupling of PSA antibody, which would cause a change in the concentration of 2DEG, and finally resulting in the slight decrease in the conductance for the device.

**Fig. 8:**
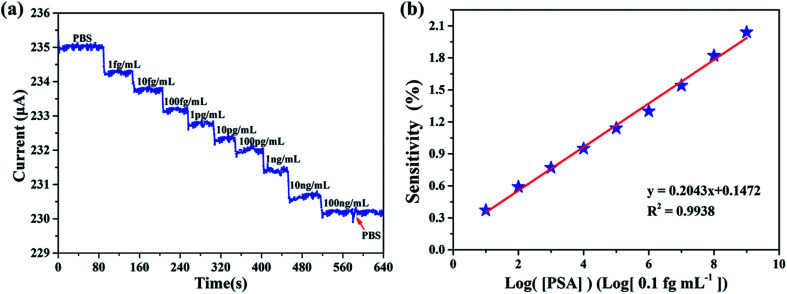
(a) Plot of the source and current *versus* time for the PSA concentration range from 1 fg mL^−1^ to 100 ng mL^−1^. (b) Linear relationship between measurement sensitivity and the logarithm of the target PSA concentrations.

Measurements were conducted by introducing PSA contained PBS buffer to the sensing membrane of AlGaN/GaN HEMT. Since PSA is negatively charged in PBS,^28^ an increase of negative charge occurred on the surface of the sensing membrane, which will cause a decrease of 2DEG concentration and eventually a source and drain current (*I*_ds_) drop at a fixed source and drain voltage (*V*_ds_). Fig. 8a illustrates the real-time detection of PSA and anti-PSA bonding in PBS buffer, it can be observed that the biosensor successfully detected the PSA for the concentration range from 1 fg mL^−1^ to 100 ng mL^−1^. Measurements indicated that the source and drain current (*I*_ds_) of biosensor decreased rapidly to a constant value of 800 nA upon addition of a 1 fg mL^−1^ PSA in PBS solution. The decrease in source and drain current (*I*_ds_) upon addition of PSA is consistent with binding of a negatively charged species to the n-type undoped AlGaN/GaN HEMT surface. It is worth noting that the limit of detection was 1 fg mL^−1^, which is lower than any previous reports to the best of our knowledge. After the last addition of 100 ng mL^−1^ PSA, PBS buffer was added into the reaction chamber. And it is obvious that the source and drain current (*I*_ds_) did not show any change, which imply that the biosensor responded only to PSA.

**Fig. 9:**
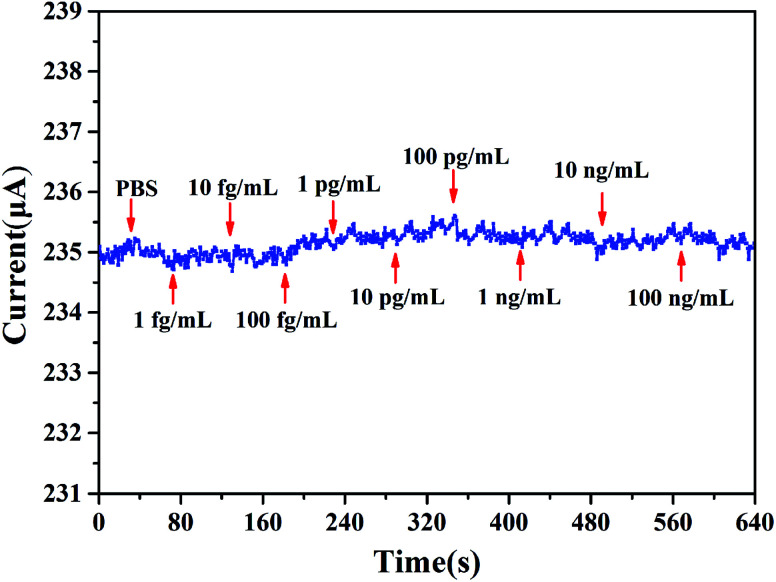
The change in the source and drain current *versus* PSA concentration from 1 fg mL^−1^ to 100 ng mL^−1^ in the absence of immobilized anti-PSA.

We define [PSA] as the concentration of PSA. The sensitivity is defined as *S* = |Δ*I*/*I*_0_| × 100%, where Δ*I* is the change of the source and drain current (*I*_ds_), and *I*_0_ means the initial current. The measurement of sensitivity as a function of PSA concentration is displayed in Fig. 8b, it can be found that the linear fitting curve has a logarithmic sensitivity as a function of PSA concentration, where the *R*^2^ = 0.9938 and the corresponding relationship of *y* = 0.2043*x* + 0.1472, the standard deviation of the test is 2.92%. The sensitivity for 100 ng mL^−1^ PSA is 2.04%. The specificity and selectivity of our biosensor is also explored by adding different concentrations of PSA to the sensing membrane without immobilized anti-PSA (BSA was also utilized to block the non-specific reactive sites), from Fig. 9 we can hardly observe the source and drain current (*I*_ds_) change no matter what concentration of PSA was added, which illustrated that non-specific binding cannot occur without anti-PSA, and the other substances in the PBS can only exert a slight influence on our biosensor. These results demonstrate that by further modification of biological antibodies on the EA modified AlGaN/GaN HEMT, our biosensors are well suited for the detection of biomolecular.

### 3.3. Discussion on the improved sensitivity of our molecular-gated AlGaN/GaN HEMT biosensors

Above we have verified the feasibility of our molecular-gated AlGaN/GaN HEMT biosensors for pH and PSA detections, next we would further explore the effect of EA modified AlGaN/GaN HEMT on the sensitivity of biosensors.

#### 3.3.1 Advantages of EA modified AlGaN/GaN HEMT for pH detection

**Fig. 10:**
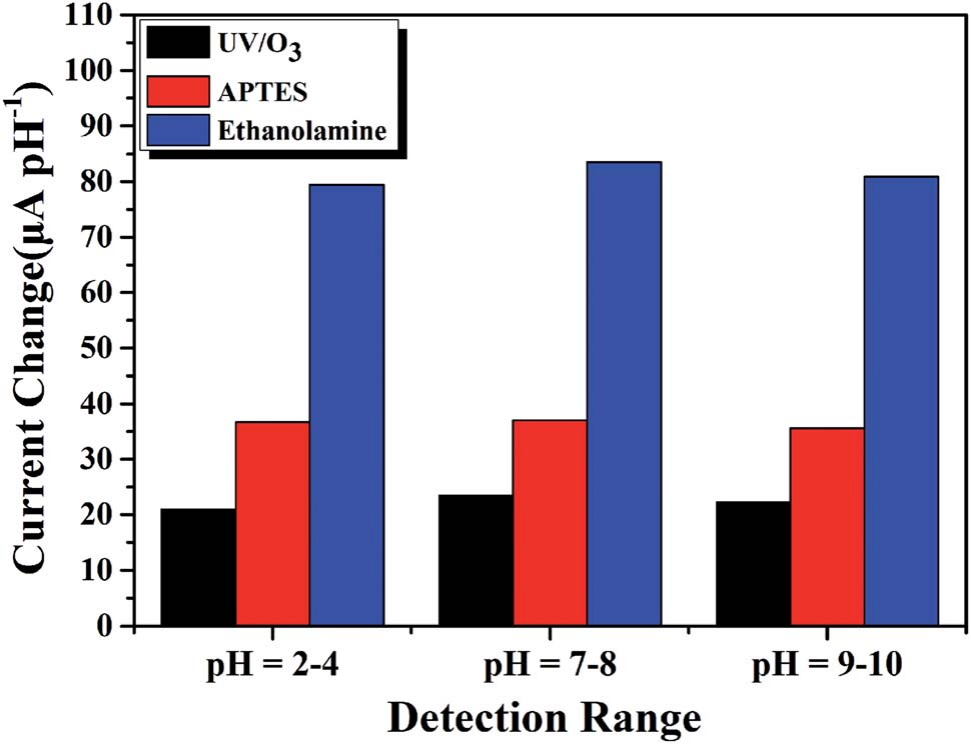
Comparison of sensing performance in the pH range of 2–4, 7–8 and 9–10 of three different kinds of AlGaN/GaN HEMT based pH sensors.

To deeply explore the improved sensitivity of our EA modified AlGaN/GaN HEMT pH sensor, we conducted pH detection experiments as follows. We utilized three kinds of AlGaN/GaN HEMT based pH sensors, traditional one where the sensing area of GaN is treated in UV/O_3_ chamber with 400 W, 20 min, molecular modified one using APTES and the one we proposed in this work using EA, to explore their pH detection performance in the range of pH = 2–4, pH = 7–8, pH = 9–10. The specific results of detection of traditional AlGaN/GaN HEMT based pH sensor is shown in Fig. S2† while of the APTES modified one is shown in Fig. S3.† To avoid unnecessary deviations, we conducted our experiments following the same steps, the results could be observed in Fig. 10.

Compare to the traditional AlGaN/GaN HEMT pH sensor, molecular modified ones regardless using APTES or EA can both provide additional amine groups which play a predominant role in pH sensing, and this explains why the APTES modified and EA modified AlGaN/GaN HEMT pH sensors show improved sensitivity. Moreover, it is obvious that EA modified AlGaN/GaN HEMT pH sensor reached a large improvement in sensitivity than APTES modified one no matter in acidic, neutral and alkaline solution. As EA modified GaN surface could provide more amine groups for pH detection owing to higher modification efficiency than APTES, which results in more active sites available to detection the variety of pH in solution.

#### 3.3.2 The improved performance of our new proposed molecular-gated AlGaN/GaN HEMT biosensors for PSA detection

The higher modification efficiency leads to more active sites for pH detection, which makes our AlGaN/GaN HEMT a better pH sensor. Here we would further discuss the improved performance of our biosensors for antigen detection. To clearly compare our new proposed molecular-gated AlGaN/GaN HEMT biosensors with those reported before, we list the corresponding comparison of the electrical parameters in Table 1.

**Table 1:** Comparison of the electrical parameters of different molecular-gated AlGaN/GaN HEMT based PSA biosensors

The structure of gate	Linear range	Limit of detection	Sensitivity for 1 ng mL^−1^	Reference
Molecular gate (using APTES for surface amination)	100 fg mL^−1^ to 1 ng mL^−1^	100 fg mL^−1^	0.215%	19
Molecular gate (using EA for surface amination)	1 fg mL^−1^ to 100 ng mL^−1^	1 fg mL^−1^	0.767%	This work

It is obvious that our molecular-gated AlGaN/GaN HEMT based biosensor using EA for surface amination show wider linear range and lower limit of detection (LOD) than reported one using APTES. The surface functionalization strategy using EA enhances drastically the surface coating quality and biomolecule detection efficiency for protein, providing thus a biocompatible surface with a highly suppressing character of nonspecific biomolecule adhesion, which explains the reason for the improved performance of our molecular-gated AlGaN/GaN HEMT biosensor. Moreover, it can be found that the sensitivity is also increased in this work, where the EA modified one exists a sensitivity of 0.767% compare to the APTES modified one of 0.215% for the detection of 1 ng mL^−1^ of PSA.

In a word, benefit from the enhanced efficiency in surface modification utilizing EA, gate region of the same area possesses more surface amine groups to detect the pH change in solution, which makes our molecular-gated AlGaN/GaN HEMT biosensor a better pH sensor. Furthermore, more surface amine groups lead to more active points for covalent coupling biological antibody for biomolecule detection, which effectively enhanced the sensitivity of our AlGaN/GaN HEMT biosensor for PSA detection. These results illustrate that using EA modification as the surface amination on GaN surface can actually enhance the sensitivity for biosensing, which demonstrates the very promising usage of EA on GaN and it is reasonable to believe that the concept of which can also be extended in many other AlGaN/GaN HEMT based biosensing directions.

## 4. Conclusion

In summary, we propose a new surface modification strategy utilizing EA for surface amination on GaN to overcome the relative low sensitivity of molecular-gated AlGaN/GaN HEMT biosensors. The feasibility of EA modified AlGaN/GaN HEMT for pH and antigen detection has been verified, and originating from the high surface modification quality on GaN, our EA modified molecular-gated AlGaN/GaN HEMT biosensors is well improved for both pH detection and antigen detection. These results reflect that the surface functionalization strategy utilizing EA on GaN surface can actually enhance the performance of AlGaN/GaN HEMT as biosensors, which is of great significance for further research on biosensing characteristic of AlGaN/GaN HEMT.

## Conflicts of interest

There are no conflicts to declare.

## Supplementary Material

RA-009-C9RA02055A-s001
