# Supplementary material for: Highly sensitive AlGaN/GaN HEMT biosensors using an ethanolamine modification strategy for bioassay applications
Source: RSC Adv. 2019 May 16;9(27):15341–9. doi: 10.1039/c9ra02055a (PMC9064203; doi:10.1039/c9ra02055a)
Supplement: RA-009-C9RA02055A-s001 [file RA-009-C9RA02055A-s001.pdf]

Electronic Supplementary Material (ESI) for RSC Advances.
This journal is © The Royal Society of Chemistry 2019

SUPPORTING INFORMATION

Highly sensitive AlGaN/GaN HEMT biosensor using ethanolamine modification strategy for bioassay applications

Zhiqi Gu,[ab] Jin Wang,[bd] Bin Miao,[bc] Lei Zhao,[bce] Xinsheng Liu, [bce] Dongmin Wu,[*ab] Jiadong Li, [*abc]

a. School of Nano Technology and Nano Bionics, University of Science and Technology of China, Hefei 230026, China

b. i-Lab, Suzhou Institute of Nano-Tech and Nano-Bionics, Chinese Academy of Sciences, Suzhou 215125, People's Republic of China

c. Key Laboratory of multifunctional nanomaterials and smart systems, Chinese Academy of Sciences, Suzhou 215125, People's Republic of China

d. The College of Materials Sciences and Engineering, Shanghai University, Shanghai 200444, China.

e. The College of Nuclear Technology and Automation Engineering, Chengdu University of Technology, Chengdu 610059, China.

E-mail: jdli2009@sinano.ac.cn

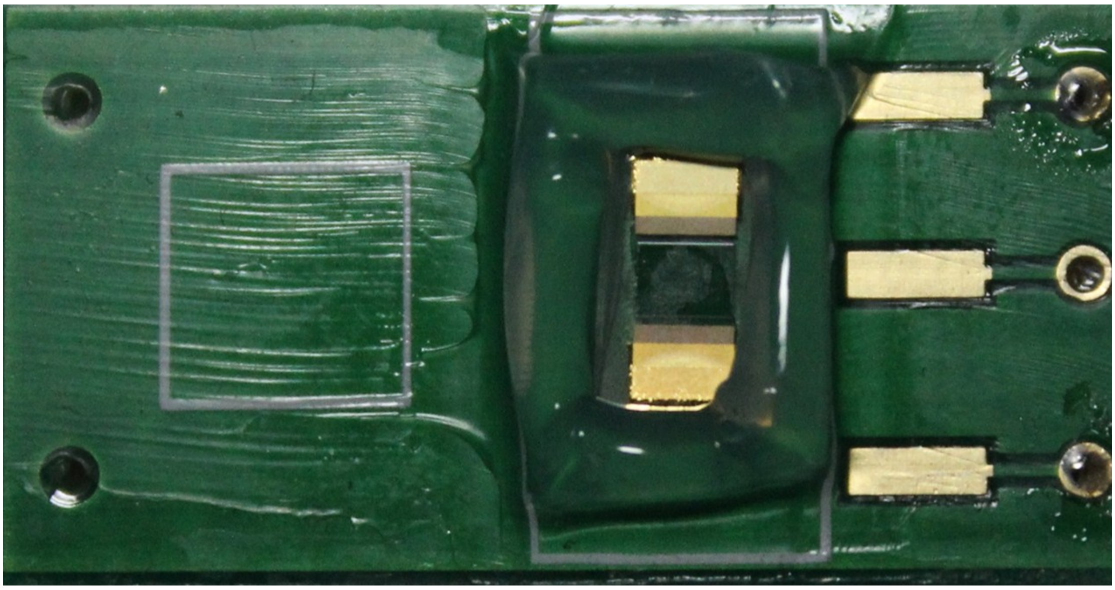

Fig. S1 The Photograph of the AlGaN/GaN HEMT biosensor chip.

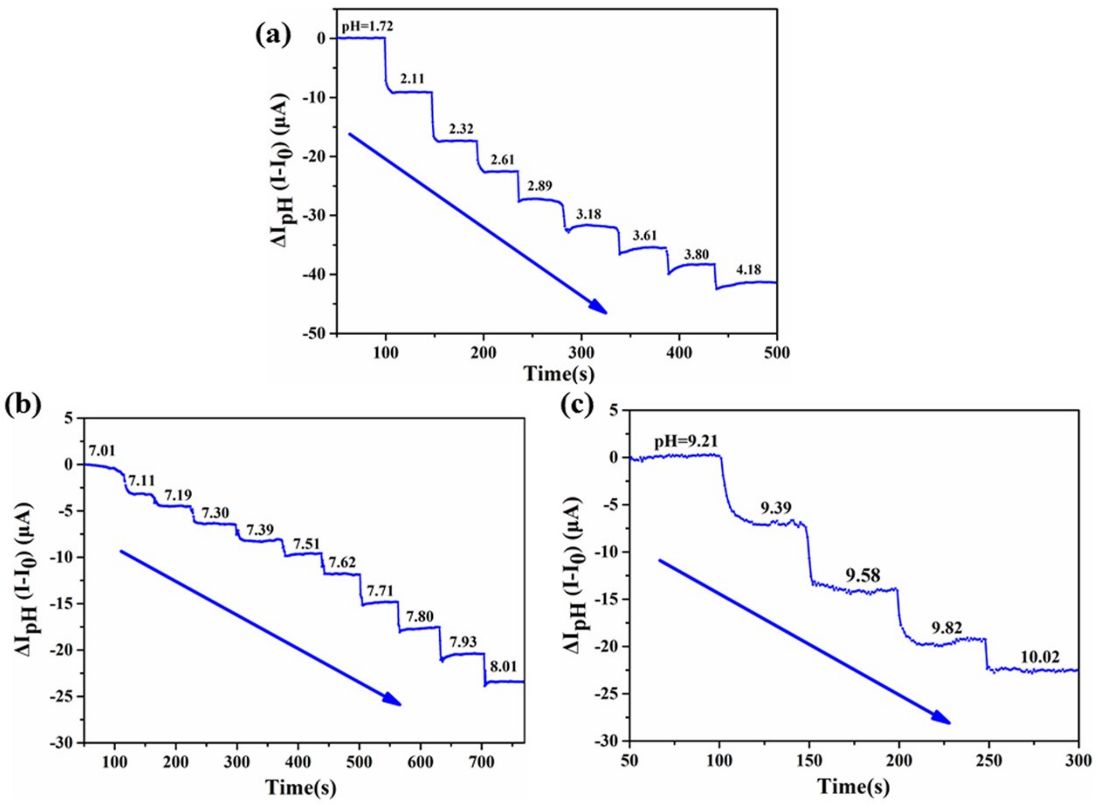

Fig. S2 Detection results of traditional AlGaN/GaN based pH sensor in the range of pH = 1.72-4.18, pH = 7.01-8.01 and pH = 9.21-10.02.

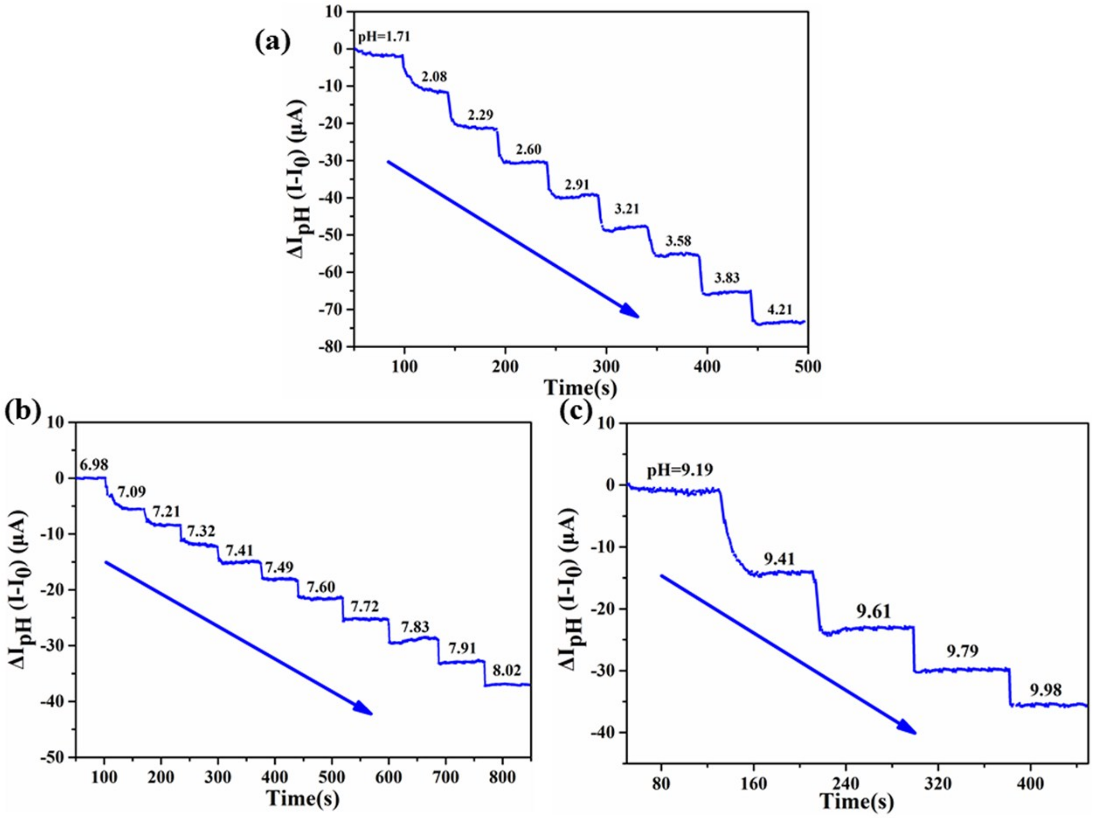

Fig. S3 Detection results of APTES modified AlGaN/GaN based pH sensor in the range of pH = 1.71-4.21, pH = 6.98-8.02 and pH = 9.19-9.98.
